# DPP-4 inhibition with linagliptin ameliorates cognitive impairment and brain atrophy induced by transient cerebral ischemia in type 2 diabetic mice

**DOI:** 10.1186/s12933-015-0218-z

**Published:** 2015-05-20

**Authors:** MingJie Ma, Yu Hasegawa, Nobutaka Koibuchi, Kensuke Toyama, Ken Uekawa, Takashi Nakagawa, Bowen Lin, Shokei Kim-Mitsuyama

**Affiliations:** Department of Pharmacology and Molecular Therapeutics, Kumamoto University Graduate School of Medical Sciences, 1-1-1 Honjo, Kumamoto, 860-8556 Japan

**Keywords:** Cerebral ischemia, Cognitive function, Brain atrophy, Oxidative stress, blood-brain barrier

## Abstract

**Background:**

It is unclear whether dipeptidylpeptidase-4 (DPP-4) inhibition can counteract the impairment of cognitive function and brain injury caused by transient cerebral ischemia in type 2 diabetes. The present study was undertaken to test our hypothesis that linagliptin, a DPP-4 inhibitor, administration following transient cerebral ischemia can ameliorate cognitive impairment and brain injury in diabetic mice.

**Methods:**

db/db mice, a model of obese type 2 diabetes, were subjected to transient cerebral ischemia by 17 min of bilateral common carotid artery occlusion (BCCAO), and were administered (1) vehicle or (2) linagliptin for 8 weeks or 1 week. For the long-term experiment on 8 weeks of linagliptin treatment, cognitive function, and volume and neuronal cell number of hippocampus and cortex were estimated in each group of mice. For the short-term experiment on 1 week of linagliptin treatment, cerebral IgG extravasation, Iba-1 positive cell number (reactive microglia), oxidative stress, and claudin-5 and gp91phox protein levels were measured in each group of mice.

**Results:**

Linagliptin administration almost completely suppressed the circulating DPP-4 activity in db/db mice, but did not significantly reduce blood glucose or ameliorate glucose intolerance in db/db mice. Linagliptin administration following transient cerebral ischemia significantly counteracted cognitive impairment in diabetic mice, as estimated by water maze test and passive avoidance test. Linagliptin administration ameliorated the decrease in cerebral volume and neuronal cell number in hippocampus and cortex of diabetic mice. Linagliptin administration significantly reduced the increase in cerebral IgG extravasation and the increase in reactive microglia caused by transient cerebral ischemia in diabetic mice. Furthermore, linagliptin significantly suppressed the increase in cerebral oxidative stress in transient cerebral ischemia-subjected diabetic mice. Furthermore, linagliptin significantly increased cerebral claudin-5 and significantly decreased gp91phox in diabetic mice subjected to transient cerebral ischemia.

**Conclusions:**

DPP-4 inhibition with linagliptin counteracted cognitive impairment and brain atrophy induced by transient cerebral ischemia in diabetic mice, independently of blood glucose lowering effect. This cerebroprotective effect of linagliptin was associated with the suppression of blood-brain barrier disruption and the attenuation of cerebral oxidative stress. Thus, our present work highlights DPP-4 inhibition as a promising therapeutic strategy for cognitive impairment and cerebral vascular complications in type 2 diabetes.

## Introduction

Type 2 diabetes is one of the major risk factors contributing to stroke, ischemic heart disease, or heart failure [1, 2], and is known to be associated with the increased risk of cognitive decline such as Alzheimer’s disease and vascular dementia [3–5]. However, there has been controversy regarding whether strict glycemic control can reduce macrovascular disease in type 2 diabetic patients. Furthermore, it remains to be determined whether glycemic control can prevent the onset or progression of cognitive impairment in diabetic patients.

Dipeptidylpeptidase 4 (DPP-4) inhibitors are a new class of blood glucose-lowering drug and used for treatment of type 2 diabetes [6–9]. DPP-4 inhibitors have low risk of hypoglycemia and neutral effect on body weight and take the advantage of less adverse events than other conventional anti-diabetic agents. DPP-4 inhibitors, through the inhibition of degradation of incretin hormone, glucagon-like peptide-1 (GLP-1), prolong the physiologic effect of GLP-1, thereby enhancing physiologically regulated insulin secretion. DPP-4 inhibitors are thought to participate in the regulation of other peptides than GLP-1, since DPP-4 is a multifunctional enzyme and cleaves a number of other substrates than GLP-1, such as the sister incretin gastric inhibitory polypeptide (GIP), neuropeptide, cytokines, and chemokines [6–9]. Thus, DPP-4 inhibitors are proposed to potentially exert pleiotropic effects independently of blood glucose lowering effect. Previous preclinical studies show that DPP-4 inhibitors counteract stroke in the normal and diabetic mouse brain [10], ameliorate cognitive impairment in streptozotosin-induced diabetic rat [11], high-fat fed mice [12], and a mouse model of chronic cerebral hypoperfusion [13], lessen the development of cerebral infarction induced by temporally focal ischemia in non-diabetic normal mice [14], improve cognition in high-fat diet induced insulin resistant rats [15], and delay some forms of Alzheimer’s disease pathology in Alzheimer’s prone mice [16]. However, it remains to be defined whether DPP-4 inhibitor administration following short transient cerebral ischemia can counteract cognitive impairment and brain injury in type 2 diabetes. In the present study, we hypothesized that DPP-4 inhibition, partially independently of blood glucose control, might ameliorate cognitive impairment and brain atrophy induced by transient cerebral ischemia in diabetic mice and if so, examined the potential role of blood-brain barrier disruption and oxidative stress. To test our hypothesis, we used db/db mice, since db/db mice is recognized as one of the most popular animal models of type 2 diabetes with obesity [17, 18], and also used linagliptin for specific DPP-4 inhibition, because linagliptin is shown to exert brain protective effect in mice independently of blood glucose control [10].

## Methods

### Animals

All procedures were carried out according to the Kumamoto University Committee for Laboratory Animal Care and use. All of the experimental procedures were performed in accordance with guidelines on animal science. Male db/db mice and nondiabetic C57BL/6 J mice were purchased from Japan Charles River Laboratories Japan Inc. (Yokohama, Japan). All animals were housed in an animal facility with a 12-hour light–dark cycle and were given the standard chow and water ad libitum.

### Drugs

Linagliptin was kindly provided by Boehringer Ingelheim.

### Experiment I protocol

**Fig. 1:**
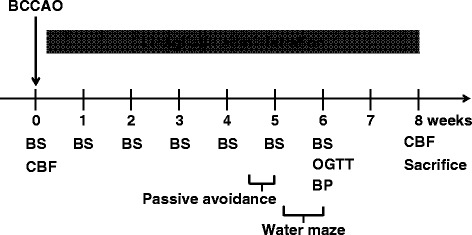
Experimental design. Abbreviations used: BCCAO, bilateral common carotid arterial occlusion; BS, blood sugar measurement; CBF, measurement of cerebral blood flow; OGTT, oral glucose tolerance test; BP, measurement of blood pressure

The detail of Experimental I protocol is shown in Fig. 1. Eight-week-old male db/db mice were subjected to 17 min of transient global cerebral ischemia, as described below. Following transient global cerebral ischemia, db/db mice were randomly assigned 2 groups, and were given (1) the standard diet (MF diet, ORIENTAL YEAST Co., Ltd, Tokyo, Japan) or (2) the standard diet containing linagliptin (0.083 g/kg diet), for 8 weeks. This dose of linagliptin (0.083 g/kg diet) was established to sufficiently inhibit the circulating DPP-4 activity [19, 20]. In our preliminary experiments, we measured diet intake of db/db mice and found that oral intake of linagliptin in our present study was roughly 10 mg/kg/day. This dose of linagliptin is regarded as an appropriate dose because 3–10 mg/kg/day of linagliptin by oral administration is most often used to examine the effect of linagliptin in vivo [10, 21, 22]. Sham-operated db/db mice were served as the control. Non-fasting blood sugar was periodically measured throughout the experiment. At specified time points, cerebral blood flow, passive avoidance test, water maze test, oral glucose tolerance test, and blood pressure measurement were performed according to the method described below (see Fig. 1). At the end of the treatment (8 weeks after BCCAO), the animals were anesthetized with 3 % isoflurane and then sacrificed for histological examination. The brain was removed quickly and cut from bregma. An 8 μm-slice was made from the level of bregma −1.43 mm to bregma −2.43 mm for the following histological evaluations including volume of hippocampus and cortex, neuronal cell number.

### Experiment II protocol

The protocol of the Experiment II was the same as that of Experiment I, except for the difference in duration of linagliptin administration. db/db mice were subjected to BCCAO for 17 min and then were given (1) the standard diet (MF diet, ORIENTAL YEAST Co., Ltd, Tokyo, Japan) or (2) the standard diet containing linagliptin (0.083 g/kg diet) for 1 week. The effect of short-term (7 days) linagliptin administration on cerebral IgG extravasation, cerebral activated microglia, and oxidative stress was examined.

### Transient global cerebral ischemia model

db/db mice were anesthetized with 3 % isoflurane and maintained with 2 % isoflurane via a facemask. The rectal temperature was controlled at 37.0 ± 0.5 °C during surgery with a feedback-regulated heating pad. Transient global ischemia was induced by bilateral common carotid artery occlusion (BCCAO). Briefly, BCCA were isolated and occluded with a microvascular clip. After 17 min, the clips were removed to allow reperfusion. The mice were kept in a single cage and allowed free access to food and water.

### Measurement of cerebral blood flow

Cerebral blood flow was measured using a laser speckle blood flow imager (Omega Zone; Omegawave, Tokyo, Japan) as previously described [23]. The blood flow at the times of pre-operation, post-operation, reperfusion and sacrifice were measured. The value of blood flow was expressed as a percentage of the pre-operation.

### Passive avoidance test

The passive avoidance test was performed as two sections including (1) training section and (2) memory test section. On the training section, we performed the test as two steps. In Step 1, we put the db/db mice into the white side of the test box and let the mice be free movement for 60 s. In Step 2, 30 min after the step one, put the mice into the white side of the test box and recorded the second until the mice went into the dark place (4 paws into the dark side). After the door was closed, the mice were given the electric shock (1.6 mA, 3 s) and kept in the dark side of the test box for 60 s. Repeat the step two until the mouse did not go into the dark side for 300 s. On the memory test section, we put the mice into the white side of the test box one day and three days after the training section, then recorded the seconds when the mouse went into the dark place (upper limit for 300 s).

### Morris water maze tests

The Morris water maze was performed as previously described [24]. The test was included four sessions: 1) training session, 2) hidden platform session, 3) visible platform test. After the training session, hidden platform test was performed five sessions per day on the following four days (day 1 to 4). On the visible platform test the platform was cued and the mice were released from different points.

### Measurement of blood pressure

The blood pressure was measured in the conscious mice at 6 weeks after BCCAO, using the tail cuff method (BP-98A; Softron Co, Tokyo, Japan).

### Oral glucose tolerance test (OGTT)

The oral glucose tolerance test was performed as previously described [25]. Briefly, mice were fasted for overnight and then orally given glucose (1 mg/g body weight). Tail vein bloods were taken and the serum glucose concentrations were measured at 0, 30, 60 and 120 min after glucose administration using a portable glucose meter (Sanwa Kagaku Kenkyusho CO., LTD, Nagoya, Japan).

### The evaluation of brain atrophy and surviving neurons

For the assessment of volume of hippocampus and cortex, the slices were selected every 200 μm from bregma −1.43 to bregma −2.43 and stained with Nissl staining. Areas of the bilateral hippocampus and cortex on each slice were calculated separately and added together and multiplied by slice thickness to give the volume. For the assessment of surviving pyramidal cells and the neurons in cortex, the selected area of hippocampus (1 field in each side of CA1 region at × 200 magnification) and cortex (2 fields were counted in each side at × 200 magnification) were counted using Lumina Vision version 2.2.0 analysis software and the average number of surviving pyramidal cells and neurons in cortex were used for evaluation of the neuronal injury.

### The measurement of activated microglia

For the assessment of number of activated microglia, brain sections were immunostained with anti-ionized calcium binding adaptor molecule-1. (Iba-1(ab5076); 1:500; Abcam, Tokyo, Japan) as previously described [26]. The number of positive cells was counted in both the CA1 region of hippocampus (1 field was counted in each side of CA1 region at × 200 magnification) and the cortex (2 fields were counted in each side at × 200 magnification). The number of cells was expressed as cells/mm^2^.

### Measurement of immunoglobulin G extravasation

Brain immunolocalization of immunoglobulin G was measured as previously described [27, 28]. IgG immunoreactivity was quantified in both sides the CA1 region of hippocampus (1 field was counted in each side of CA1 region at × 200 magnification) and the cortex (2 fields were counted in each side of cortex at × 200 magnification). The value was measured with Lumina Vision version 2.2.0 analysis software (Mitani Corporation, Tokyo, Japan) and expressed as the mean value compared with the sham-operated group.

### Detection of superoxide

To detect superoxide levels in hippocampus and cortex, dihydroethidium (DHE; SIgam, St. Louis, MO, USA) staining was used in situ as previously described [29]. The frozen tissue sections were stained with DHE fluorescence and measured using Lumina Vision version 2.2.0 analysis software. The mean fluorescence was quantified in both sides the CA1 region of hippocampus (1 field was counted in each side of CA1 region at × 200 magnification) and the cortex (2 fields were counted in each side of cortex at × 200 magnification). Each value was expressed related to individual group sham-operated mouse. The results were expressed relative to values obtained for sham-operated mouse.

### Western blotting

The detailed Western blot method has been previously described [30]. Briefly, the front part of brain protein extracts were subjected to sodium dodecyl sulfate-polyacrylamide gel electrophoresis and transferred to polyvinylidene difluoride membranes. The antibodies used were as follows: anti-claudin-5 (ab53765) (1:500; Abcam), GAPDH (sc-32233) (1:5000; Santa Cruz Biotechnology, Santa Cruz, CA, USA), anti-gp91phox (sc-20782) (1:1000; Santa Cruz Biotechnology, Santa Cruz, CA, USA). The membranes were incubated for 1 h with appropriate secondary antibodies and processed with an enhanced chemiluminescence reagent kit (Amersham ECL plus kit, GE Healthcare, UK). Mouse or rabbit secondary antibody (1:5000 or 1:30000; Cell Signaling) was used in accordance with first antibody. The antibodies were visualized by using an enhanced chemiluminescence method (ECL Plus; GE Healthcare, Buckinghamshire, UK). The intensity of the bands was quantified by using analysis software (Image J; National Institute of Health, Bethesda, MD, USA). In individual samples, each value was corrected for that of GAPDH.

#### Measurement of DPP-4 activity and GLP-1 in serum and brain tissue and serum insulin

DPP-4 activity, GLP-1 concentrations, and serum insulin were determined in samples from mice subjected to 8 weeks of linagliptin treatment. Serum and brain DPP-4 activities were measured using DPP4-Glo Protease Assay (Promega, Madison, WI, USA), as described [21]. Active GLP-1 concentrations in serum and brain tissue were measured by the Active form Assay Kit-IBL (Immuno-Biological Laboratories, Gunma, Japan). Serum insulin concentrations were measured by Morinaga ultra sensitive mouse insulin ELISA kit.

#### Statistical analysis

Data were presented as mean ± SEM. The data on time course experiments were analyzed by two-way ANOVA with repeated measures followed by Bonferroni post hoc test for multiple comparisons. Statistical significance was determined with one-way ANOVA, followed by the Tukey's multiple comparisons test between each group. When a normal distribution was not confirmed among comparison groups, data were analyzed by Dunn’s multiple comparison test. Data were analyzed by non-paired t-test when two groups were compared. All analyses were performed using GraphPad Prism 6.0 for Mac (GraphPad Software, Inc; La Jolla, CA, USA). In all tests, differences were considered statistically significant at a value of P < 0.05.

## Results

### Effect of transient BCCAO on non-diabetic versus diabetic mice

**Fig. 2:**
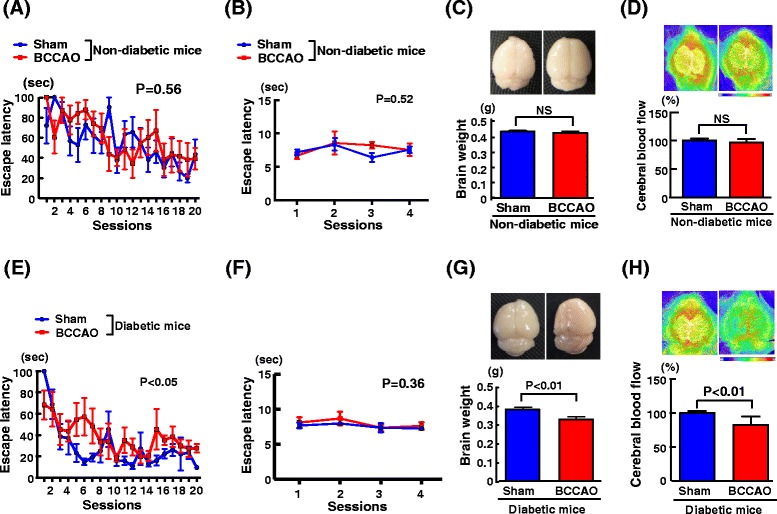
Effect of transient cerebral ischemia on cognitive function and brain injury in non-diabetic mice versus diabetic mice. Cognitive function was estimated by Morris water maze test. **a**, **b**, **c**, and **d** indicate escape latency of the hidden platform test on 4 consecutive days (1–20 sessions), escape latency in the visible test, brain weight, and cerebral blood flow, respectively, in non-diabetic mice. Each value represents mean ± SEM (n = 6 in Sham, n = 5 in BCCAO). **e**, **f**, **g**, and **h** indicate escape latency of the hidden platform test on 4 consecutive days (1–20 sessions), escape latency in the visible test, brain weight, and cerebral blood flow, respectively, in diabetic mice. Each value represents mean ± SEM (n = 6 in Sham, n = 5 in BCCAO). Abbreviations used: Sham, sham-operated group; BCCAO, group subjected to bilateral common carotid arterial occlusion

In our preliminary experiment, we compared the effect of transient global cerebral ischemia (17 min of BCCAO) between non-diabetic and diabetic mice. As shown in Fig. 2a-d, 17 min of BCCAO did not significantly impair cognitive function and visual acuity, and did not affect brain weight or cerebral blood flow in non-diabetic mice, compared with sham operation. In contrast to non-diabetic mice, 17 min of BCCAO significantly impaired cognitive function and significantly decreased brain weight and cerebral blood flow in diabetic db/db mice (Fig. 2e-h).

### Effect of linagliptin on body weight, blood sugar, glucose tolerance, insulin concentrations, DPP-4 activity, GLP-1 concentrations, and blood pressure of db/db mice subjected to transient BCCAO

**Fig. 3:**
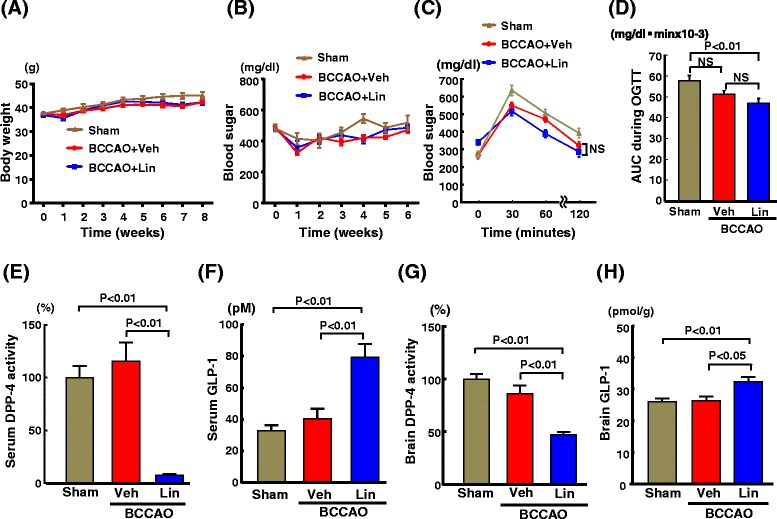
Effect of long-term linagliptin administration on body weight (**a**), blood sugar (**b**), oral glucose tolerance test (**c**), blood sugar area under curve (AUC) during OGTT (**d**), serum DPP-4 activity (**e**), serum GLP-1 (**f**), brain DPP-4 activity (**g**), and brain GLP-1 (**h**) in db/db mice subjected to transient BCCAO. OGTT in (**c**) and (**d**) were performed after 6 weeks of linagliptin administration. In (**e**), the mean value of sham group was expressed as 100 %. Abbreviations used: Sham, sham-operated group; BCCAO + Veh, group subjected to transient BCCAO followed by vehicle administration; BCCAO + Lin, group subjected to transient BCCAO followed by linagliptin administration; AUC, area under curve; NS, not significant between groups. Each value represents mean ± SEM. In (**a**)-(**d**), n = 10 in Sham, n = 10 in BCCAO + Veh, n = 11 in BCCAO + Lin. In (**e**) and (**f**), n = 9 in Sham, n = 5 in BCCAO + Veh, n = 7 in BCCAO + Lin. In (**g**) and (**h**), n = 10 in Sham, n = 9 in BCCAO + Veh, n = 11 in BCCAO + Lin

Eight weeks of linagliptin administration (Fig. 3a) did not significantly alter body weight in db/db mice subjected to transient BCCAO. There was no significant difference in blood sugar throughout the treatment between linagliptin and vehicle groups of db/db mice subjected to transient BCCAO (Fig. 3b). Furthermore, as shown by OGTT and blood sugar area under curve (AUC) during OGTT in Fig. 3c and d, linagliptin did not significantly improve glucose tolerance in db/db mice subjected to transient BCCAO. However, blood sugar AUC in linagliptin group was significantly smaller than that in sham group (P < 0.01). Serum insulin concentrations in sham group, vehicle group subjected to BCCAO, and linagliptin group subjected to BCCAO were 1.62 ± 0.51, 1.16 ± 0.29, and 1.08 ± 0.21 ng/ml, respectively, and there was no significant difference in serum insulin concentrations among the groups.

As shown in Fig. 3e, serum DPP-4 activity in linagliptin-treated group subjected to BCCAO was much less than that in vehicle-treated group subjected to BCCAO (P < 0.01) or sham-operated group (P < 0.01). Serum GLP-1 concentrations in linagliptin-treated group were significantly larger than those in vehicle group or sham group (Fig. 3f). As shown in Fig. 3g and h, linagliptin treatment significantly decreased brain DPP-4 activity (P < 0.01) and significantly increased brain GLP-1 concentrations (P < 0.05) in db/db mice subjected to BCCAO compared with vehicle treatment.

Blood pressure was 129 ± 4 mmHg in sham group, 110 ± 3 mmHg in vehicle group subjected to transient BCCAO, and 117 ± 2 mmHg in linagliptin group subjected to transient BCCAO, and there was no significant difference in blood pressure between vehicle and linagliptin groups of db/db mice.

### Effect of linagliptin on cognitive function of db/db mice subjected to transient BCCAO

**Fig. 4:**
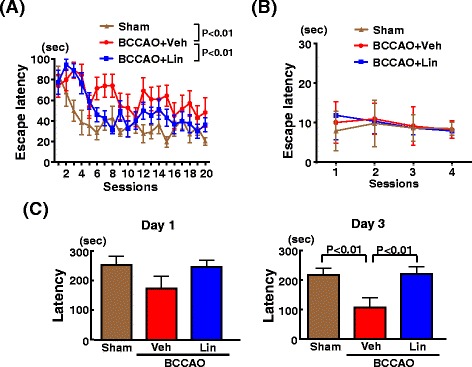
Effect of long-term linagliptin administration on escape latency of the hidden platform test (**a**), escape latency in the visible test (**b**), latency of the passive avoidance test (**C**) in db/db mice subjected to transient BCCAO. Abbreviations used are the same as in Fig. 4. Each value represents mean ± SEM (n = 10 in Sham, n = 10 in BCCAO + Veh, n = 11 in BCCAO + Lin)

As shown by Fig. 4a, transient BCCAO of db/db mice significantly increased escape latency of the hidden platform test on water maze test (P < 0.01) compared with sham operation. Linagliptin administration significantly suppressed the increase in escape latency of the hidden platform test in db/db mice subjected to BCCAO (P < 0.01). In Fig. 4b, there was no significant difference in visual acuity between the groups. As shown in Fig. 4c, transient BCCAO in db/db mice significantly decreased latency on day 3 of passive avoidance test compared with sham operation (P < 0.01) and this decrease in latency of db/db mice subjected to BCCAO was significantly suppressed by linagliptin (P < 0.01).

### Effect of linagliptin on cerebral blood flow and brain weight of db/db mice subjected to transient BCCAO

**Fig. 5:**
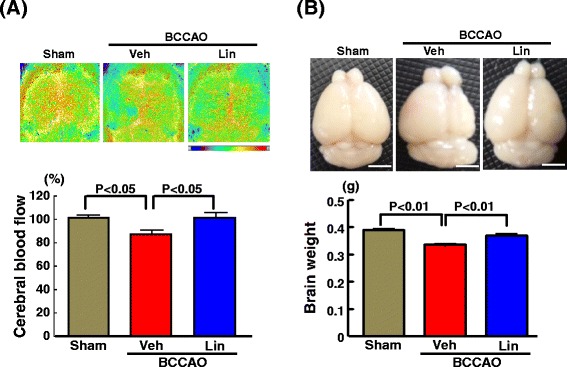
Effect of long-term linagliptin administration on cerebral blood flow (**a**) and brain weight (**b**) in db/db mice subjected to transient BCCAO. Abbreviations used are the same as in Fig. 4. Each value represents mean ± SEM (n = 10 in Sham, n = 10 in BCCAO + Veh, n = 11 in BCCAO + Lin). Upper panels in (**a**) and (**b**) indicate representative cerebral blood flow image and whole brain, respectively of each group of mice. Bar = 3 mm

As shown in Fig. 5, being in consistent with our preliminary experiment (Fig. 2), transient BCCAO in db/db mice significantly decreased cerebral blood flow (P < 0.05) and brain weight (P < 0.01) compared with sham operation. Linagliptin administration significantly suppressed the decrease in cerebral blood flow (P < 0.05) and the decrease in brain weight (P < 0.01) of db/db mice subjected to BCCAO.

### Effect of linagliptin on volume and neuronal cell number of hippocampus and cortex of db/db mice subjected to transient BCCAO

**Fig. 6:**
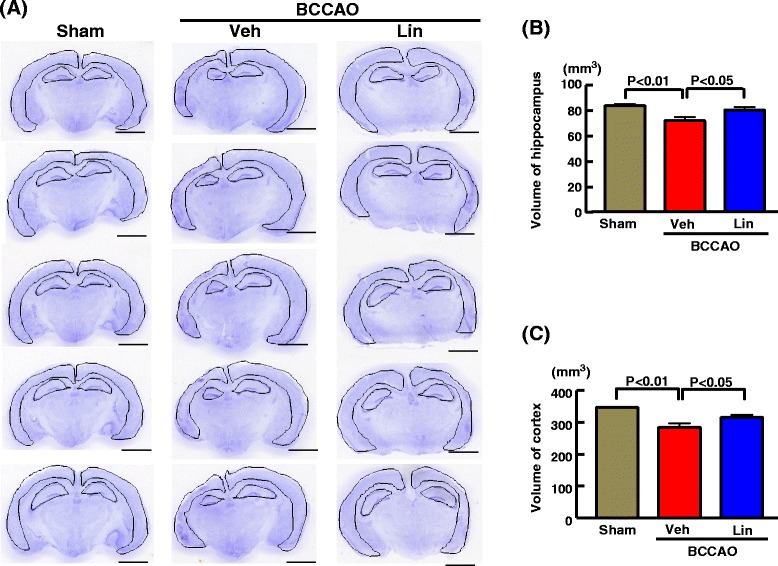
Effect of long-term linagliptin administration on volume of hippocampus and cortex in db/db mice subjected to transient BCCAO. **a** indicates representative image of Nissle-stained cerebral coronal section of each group. Bar = 2 mm. **b** and **c** indicate volume of hippocampus and cortex, respectively of each group. Abbreviations used are the same as in Fig. 4. Each value represents mean ± SEM (n = 10 in Sham, n = 10 in BCCAO + Veh, n = 11 in BCCAO + Lin)

As shown in Fig. 6, transient BCCAO in db/db mice significantly reduced volume of hippocampus (P < 0.01) and volume of cortex (P < 0.01) compared with sham operation. Linagliptin administration ameliorated the decrease in volume of hippocampus (P < 0.05) or volume of cortex (P < 0.05) of BCCAO-subjected db/db mice.

**Fig. 7:**
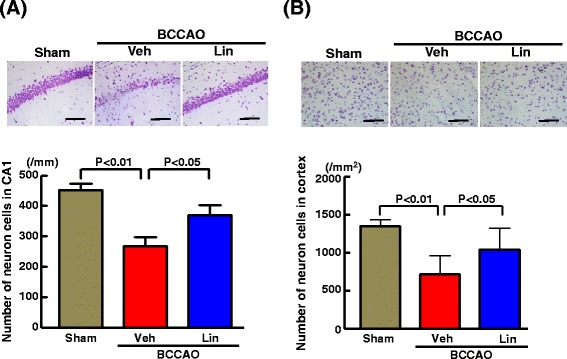
Effect of long-term linagliptin administration on number of neuronal cells in hippocampal CA1 region (**a**) and cortex (**b**) in db/db mice subjected to transient BCCAO. Upper panels in (**a**) and (**b**) indicate representative photomicrographs of hippocampus and cortex, respectively, of each group. Bar = 100 μm. Abbreviations used are the same as in Fig. 4. Each value represents mean ± SEM ((**a**): n = 10 in Sham, n = 10 in BCCAO + Veh, n = 11 in BCCAO + Lin. **b**: n = 7 in Sham, n = 6 in BCCAO + Veh, n = 7 in BCCAO + Lin)

As shown in Fig. 7, transient BCCAO in db/db mice significantly reduced number of neuronal cells in hippocampal CA1 region (P < 0.01) and in cortex (P < 0.01). Linagliptin administration significantly prevented the reduction of neuronal cell number in hippocampal CA1 (P < 0.05) and in cortex (P < 0.05) of BCCAO-subjected db/db mice.

### Effect of 7 days of linagliptin administration on blood-brain barrier, reactive microglia, and cerebral oxidative stress of db/db mice subjected to transient BCCAO

**Fig. 8:**
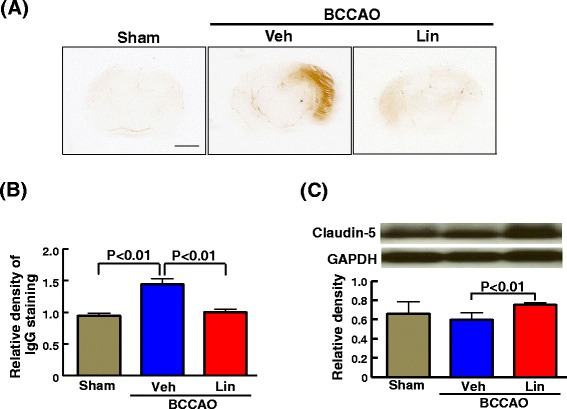
Effect of 7 days of linagliptin administration on IgG extravasation and cerebral claudin-5 in the brain of db/db mice subjected to transient BCCAO. **a** indicates representative image of IgG immunostained cerebral section of each group and (**b**) indicates relative density of IgG staining. Bar = 2 mm. (**c**) indicates relative density of cerebral claudin-5 estimated by western blot. Upper panels in (**c**) indicate representative western blot band. In individual mouse, claudin-5 wes corrected for GAPDH. Abbreviations used are the same as in Fig. 3. Each value represents mean ± SEM (n = 7 in Sham, n = 6 in BCCAO + Veh, n = 7 in BCCAO + Lin.)

As shown in Fig. 8a and b, transient BCCAO in db/db mice significantly increased IgG extravasation in the brain compared with sham operation (P < 0.01) and this increase in IgG extravasation was significantly ameliorated by linagliptin (P < 0.01). As shown in Fig. 8c, linagliptin administration to BCCAO-subjected db/db mice significantly increased cerebral claudin-5 (P < 0.01).

**Fig. 9:**
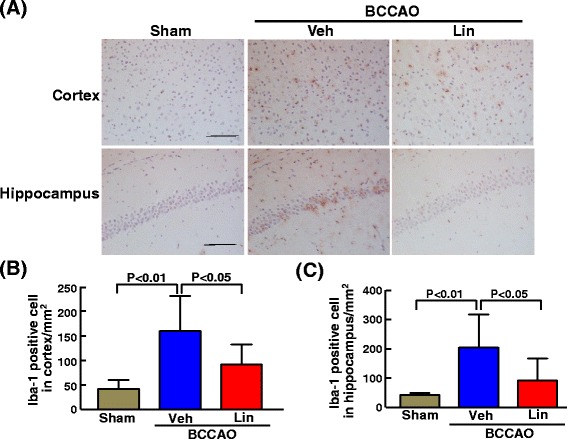
Effect of 7 days of linagliptin administration on Iba-1 positive cell number in cortex and hippocampus of db/db mice subjected to transient BCCAO. **a** indicates representative photomicrograph of Iba-1 immunostained cerebral sections. Bar = 100 μm. **b** and **c** indicate Iba-1 positive cell number in cortex and hippocampus, respectively. Abbreviations used are the same as in Fig. 4. Each value represents mean ± SEM (n = 7 in Sham, n = 6 in BCCAO + Veh, n = 7 in BCCAO + Lin.)

As shown in Fig. 9, transient BCCAO significantly increased Iba-1 positive cell number in cortex (P < 0.01) and in hippocampus (P < 0.01) of db/db mice, and the increase in Iba-1positive cell number induced by BCCAO was significantly suppressed by linagliptin administration.

**Fig. 10:**
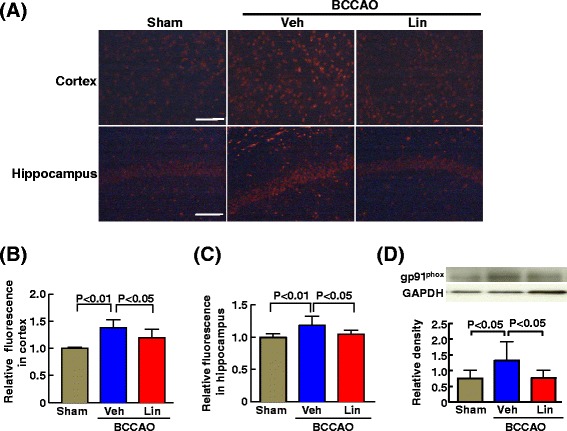
Effect of 7 days of linagliptin administration on oxidative stress in cortex and hippocampus, and cerebral gp91phox of db/db mice subjected to transient BCCAO. **a** indicates representative photomicrograph of DHE-stained cerebral sections. Bar = 100 μm. **b** and **c** indicate relative DHE fluorescence in cortex and hippocampus, respectively. **d** indicates cerebral gp91phox levels corrected to GAPDH. In **d**, upper panels indicate representative western blot band. Abbreviations used are the same as in Fig. 3. Each value represents mean ± SEM (n = 7 in Sham, n = 6 in BCCAO + Veh, n = 7 in BCCAO + Lin.)

DHE staining of cerebral sections in Fig. 10a-c showed that oxidative stress in cortex and hippocampus was greater in BCCAO-subjected db/db mice than in sham group of db/db mice. Linagliptin administration to BCCAO-subjected db/db mice significantly reduced oxidative stress in cerebral cortex (P < 0.05) and in hippocampus (P < 0.05). As shown in Fig. 10d, linagliptin administration to BCCAO-subjected db/db mice significantly decreased cerebral gp91phox (P < 0.05).

## Discussion

The purpose of the present study was to test our hypothesis that DPP-4 inhibition with linagliptin following short transient cerebral ischemia can counteract cognitive impairment and brain atrophy in obese type 2 diabetes. The major findings of this study were that linagliptin administration counteracted the impairment of cognitive function and brain atrophy induced by short transient cerebral ischemia in type 2 diabetic mice and this cerebroprotective effect of linagliptin was associated with the suppression of blood-brain barrier disruption and the attenuation of oxidative stress in the brain of diabetic mice.

The duration of cerebral ischemia by BCCAO used in this study was short time (for 17 min) and did not significantly cause impairment of cognitive function and occurrence of brain atrophy in non-diabetic mice. Interestingly, in contrast to non-diabetic mice, such a short transient cerebral ischemia significantly impaired cognitive function and caused brain atrophy in diabetic mice. Therefore, it is possible that hyperglycemia might enhance the vulnerability to ischemic brain injury. However, the potential role of obesity and hyperlipidemia in ischemic brain injury cannot be excluded, since db/db mice exhibit not only hyperglycemia but also obesity and hyperlipidemia [18, 31]. Of note, linagliptin administration following transient cerebral ischemia in diabetic mice significantly decreased escape latency of the hidden platform test on water maze test and significantly increased latency on passive avoidance test, providing the evidence that linagliptin ameliorated cognitive dysfunction induced by transient cerebral ischemia in diabetic mice. Furthermore, linagliptin administration significantly ameliorated the decrease in cerebral blood flow and the decrease in brain weight, and these effects of linagliptin were associated with the suppression of the decrease in volume of hippocampus and cortex and with the inhibition of decrease in hippocampal and cortical neuronal cell number. These results demonstrate that linagliptin suppressed cerebral ischemia-induced brain atrophy in diabetic mice, through the inhibition of neuronal cell death.

blood-brain barrier plays a key role in the pathophysiology of brain function, stroke, and dementia [32–35]. The disruption of blood-brain barrier causes the enhancement of cerebral oxidative stress and inflammation. Furthermore, oxidative stress and inflammation form vicious cycle and accelerates brain injury and cognitive impairment. To address the potential mechanism underlying the above mentioned brain protective effects of linagliptin, we examined the effects of linagliptin administration on blood-brain barrier function, activation of microglia, and oxidative stress in transient cerebral ischemia-induced diabetic mice. Linagliptin administration significantly ameliorated the disruption of blood-brain barrier and the activation of microglia, as shown by the reduction of cerebral IgG extravasation and by the reduction of Iba-1 positive cell number by linagliptin in diabetic mice subjected to cerebral ischemia. Furthermore, linagliptin significantly attenuated cerebral oxidative stress induced by cerebral ischemia in diabetic mice, as shown by the significant attenuation of superoxide in hippocampal and cortical tissues by linagliptin. To address the underlying molecular mechanism, we examined the effect of linagliptin on cerebral claudin-5 and gp91phox levels in cerebral ischemia-subjected diabetic mice, since claudin-5 [36, 37] is a main cerebral endothelial tight junction protein which plays a major role in blood-brain barrier function and gp91phox is a major subunit of NADPH oxidase, the main oxidative stress-generating enzyme. We found that linagliptin significantly increased cerebral claudin-5 and decreased the increase in gp91phox in diabetic mice. Hence, the prevention of blood-brain barrier disruption and the attenuation of cerebral oxidative stress by linagliptin seem to be partially mediated by the increase in claudin-5 and the decrease in gp91phox, respectively. However, further study is needed to define the detailed molecular mechanism responsible for brain protective effects of linagliptin in transient cerebral ischemia-subjected diabetic mice.

It is a very critical issue as to whether the cerebroprotective effects of linagliptin observed in this study was attributed to glucose lowering effect or not. In the present study, linagliptin treatment almost completely suppressed serum DPP-4 activity and significantly increased serum GLP-1 levels in db/db mice. Moreover, linagliptin treatment significantly decreased brain DPP-4 activity and increased brain GLP-1 concentrations in db/db mice. These results confirm that the dose of linagliptin (0.083 g/kg diet) used in this study was pharmacologically enough dose. However, sufficient suppression of DPP-4 activity led to only small improvement of glucose tolerance in db/db mice. Our present findings are in good agreement with the previous reports showing that marked DPP-4 inhibition failed to reduce blood glucose or improve glucose tolerance in db/db mice with similar ages to our present work [22, 38, 39]. In contrast to no significant improvement of glucose tolerance by DPP-4 inhibition in db/db mice at the late stage of severe diabetes [22, 38, 39], linagliptin is reported to significantly reduce blood glucose and improve glucose tolerance in younger db/db mice at the early stage of diabetes [40]. Collectively, our present findings showing only slight improvement of glucose tolerance with sufficient DPP-4 inhibition can be explained by the use of older db/db mice with severe insulin resistance and diabetes. Despite slight improvement of glucose tolerance, DPP-4 inhibition with linagliptin significantly protected against brain injury in db/db mice subjected to BCCAO. Furthermore, GLP-1 receptor activation is shown to exert neuroprotection and protect against cognitive impairment in mice [41]. Collectively, our preset work provided the notion that linagliptin exerts brain protective effects partially via blood glucose-independent mechanism. However, it cannot be ruled out that brain protective effects of linagliptin might be partially mediated by blood glucose control, since linagliptin slightly but significantly improved glucose tolerance in db/db mice.

### Study limitations

There are several study limitations in our present work. First, in the present study, cerebral claudin-5 levels did not differ between sham and vehicle groups, thereby not excluding the possibility that clauding-5 might play a minor role in blood-brain barrier disruption induced by BCCAO. Further study is necessary for the precise mechanism of blood-brain barrier disruption in this study. Second, in the present study, we did not measure GIP levels in db/db mice, because the pathophysiological role of GIP elevation by DPP-4 inhibition is poorly understood. Therefore, further study investigating the potential role of GIP is needed to define the precise mechanism underlying brain protective effect of linagliptin. Third, the mechanism underlying brain protection by linagliptin was examined at 1 week after BCCAO, since the significant disruption of blood-brain barrier and the enhancement of cerebral oxidative stress occurred at 1 week after BCCAO. However, the study at additional time point might be necessary for further determination of the underlying mechanism. Fourth, in the present study, we did not perform power calculation for determination of number of animals in each experimental group. However, we performed appropriate statistical analysis, thereby validating our present results. Finally, the present study did not allow us to elucidate which dose of linagliptin is appropriate to protect against stroke or cognitive impairment in clinical practice. Future randomized clinical study is required to elucidate this point.

In conclusion, our present work provided the evidence that DPP-4 inhibition with linagliptin following transient cerebral ischemia ameliorates blood-brain barrier breakdown and oxidative stress, and thereby counteracts the impairment of cognitive function and brain atrophy due to the reduction of hippocampal and cortical neuronal cell in type 2 diabetic mice. Furthermore, this cerebroprotective effect of linagliptin seems to be at least partially mediated by blood glucose-independent effect. Therefore, we propose that DPP-4 inhibition may be a promising strategy for prevention and treatment of ischemic brain injury and cognitive decline in type 2 diabetes. However, future randomized clinical study is warranted to elucidate our proposal.

## Abbreviations

DPP-4: Dipeptidylpeptidase-4
GLP-1: Glucagon-like peptide-1
